# Biclustered Independent Component Analysis for Complex Biomarker and Subtype Identification from Structural Magnetic Resonance Images in Schizophrenia

**DOI:** 10.3389/fpsyt.2017.00179

**Published:** 2017-09-26

**Authors:** Cota Navin Gupta, Eduardo Castro, Srinivas Rachkonda, Theo G. M. van Erp, Steven Potkin, Judith M. Ford, Daniel Mathalon, Hyo Jong Lee, Bryon A. Mueller, Douglas N. Greve, Ole A. Andreassen, Ingrid Agartz, Andrew R. Mayer, Julia Stephen, Rex E. Jung, Juan Bustillo, Vince D. Calhoun, Jessica A. Turner

**Affiliations:** ^1^The Mind Research Network, Albuquerque, NM, United States; ^2^Department of Biosciences and Bioengineering, Indian Institute of Technology, Guwahati, India; ^3^Computational Biology Center, IBM Thomas J. Watson Research, Yorktown Heights, NY, United States; ^4^Department of Psychiatry and Human Behavior, School of Medicine, University of California, Irvine, Irvine, CA, United States; ^5^Department of Psychiatry, School of Medicine, University of California, San Francisco, San Francisco, CA, United States; ^6^Divisions of Electronics and Information Engineering, Chonbuk National University, Jeonju, South Korea; ^7^Department of Psychiatry, University of Minnesota, Minneapolis, MN, United States; ^8^MGH/MIT/HMS Athinoula A. Martinos Center for Biomedical Imaging, Charlestown, MA, United States; ^9^NORMENT, KG Jebsen Center for Psychosis Research, Institute of Clinical Medicine, University of Oslo, Oslo, Norway; ^10^Division of Mental Health and Addiction, Oslo University Hospital, Oslo, Norway; ^11^Department of Clinical Neuroscience, Karolinska Institute, Stockholm, Sweden; ^12^Department of Research, Diakonhjemmet Hospital, Oslo, Norway; ^13^Department of Neurosurgery, University of New Mexico Health Sciences Center, Albuquerque, NM, United States; ^14^Department of Psychiatry, University of New Mexico, Albuquerque, NM, United States; ^15^Department of Electrical and Computer Engineering, University of New Mexico, Albuquerque, NM, United States; ^16^Department of Psychology, Neuroscience Institute, Georgia State University, Atlanta, GA, United States

**Keywords:** gray matter concentration, biclustering, independent component analysis, subtypes, positive and negative syndrome scale symptoms, group information-guided independent component analysis

## Abstract

Clinical and cognitive symptoms domain-based subtyping in schizophrenia (Sz) has been critiqued due to the lack of neurobiological correlates and heterogeneity in symptom scores. We, therefore, present a novel data-driven framework using biclustered independent component analysis to detect subtypes from the reliable and stable gray matter concentration (GMC) of patients with Sz. The developed methodology consists of the following steps: source-based morphometry (SBM) decomposition, selection and sorting of two component loadings, subtype component reconstruction using group information-guided ICA (GIG-ICA). This framework was applied to the top two group discriminative components namely the insula/superior temporal gyrus/inferior frontal gyrus (I-STG-IFG component) and the superior frontal gyrus/middle frontal gyrus/medial frontal gyrus (SFG-MiFG-MFG component) from our previous SBM study, which showed diagnostic group difference and had the highest effect sizes. The aggregated multisite dataset consisted of 382 patients with Sz regressed of age, gender, and site voxelwise. We observed two subtypes (i.e., two different subsets of subjects) each heavily weighted on these two components, respectively. These subsets of subjects were characterized by significant differences in positive and negative syndrome scale (PANSS) positive clinical symptoms (*p* = 0.005). We also observed an overlapping subtype weighing heavily on both of these components. The PANSS general clinical symptom of this subtype was trend level correlated with the loading coefficients of the SFG-MiFG-MFG component (*r* = 0.25; *p* = 0.07). The reconstructed subtype-specific component using GIG-ICA showed variations in voxel regions, when compared to the group component. We observed deviations from mean GMC along with conjunction of features from two components characterizing each deciphered subtype. These inherent variations in GMC among patients with Sz could possibly indicate the need for personalized treatment and targeted drug development.

## Introduction

Subtype staging using clinical features (1, 2), cognitive factors (3–5), and brain morphometry measures (6) have been attempted to characterize the heterogeneity in patients with schizophrenia (Sz) with mixed views in the research community. Univariate voxel-based morphometry (VBM) (7–10) and multivariate source-based morphometry (SBM) (11–13) are two widely used techniques to analyze structural magnetic resonance images (sMRI) differences between healthy controls (Ct) and Sz. Studies in Sz using both these techniques have reported largest (in terms of effect size) gray matter concentration (GMC) deficits for regions of left insular cortex, left inferior frontal gyrus, superior temporal gyrus, and precentral gyrus. VBM does not utilize any information about the relationships among voxels, while the SBM framework which uses an independent component analysis (ICA) module (14) provides a way to pool information across different voxels, thereby identifying common components of variation (13).

Voxel-based morphometry studies (15, 16) have used factor analysis on clinical features to divide their Sz samples into three subtypes with predominantly negative, disorganization, and paranoid symptom profiles. These studies then go on to illustrate, the considerable heterogeneity of spatial distribution and extent of structural deficits across the three Sz subtypes. This three-factor subtyping based on clinical features was also reported in chronic and old-age populations (17). From a different viewpoint, factor analysis of psychopathology ratings were found to be related to different patterns of cerebral blood flow (18). However, usage of clinical symptoms in these studies has been criticized for temporal instability and lack of neurobiological correlates (4, 19, 20). Cognitive measures in contrast may be more stable (4, 21–24) but are not the determining characteristics of the disorder. Most of the above studies first perform factor analysis on clinical or cognitive symptoms to decipher subtypes and then do VBM analysis on sMRI data having obtained the subtype grouping. Our work takes a different approach and obtains subtype grouping from the stable and reliable sMRI data and then moves to clinical symptom domain to confirm these observed subtype differences.

Few neuroimaging studies in Sz have ignored these variations in clinical and cognitive symptoms among Sz cohort, looking only at the differences in average effects between Ct and Sz (9–13, 25). Numerous review studies have pointed to varying regions of aberrations or inconsistencies in terms of gray matter, whole brain volume, and white matter differences (12, 26–28). Recent studies seem to suggest that this underlying clinical heterogeneity in Sz could be deciphered from the more reliable and stable genetic (29) and neuroimaging data (30) rather than clinical and cognitive features. These studies suggested that regional hidden local components, linked to specific clinical symptoms could exist in a subject by voxel matrix (i.e., voxel representing either GMC, fractional anisotropy (FA), or gray matter volume) or in a subject by single nucleotide polymorphism matrix depending on the spectrum of Sz participants recruited in a given study. The idea of finding complex biomarkers (lower or higher GMC in multiple regions) for subtypes of subjects in neuroimaging and the inability of univariate methods to find the underlying differences was clearly illustrated in a review article (31). It, therefore, becomes imperative to develop data-driven frameworks that can reliably decipher these complex hidden local components corresponding to various subtypes. Non-negative matrix-based biclustering methods (32, 33) have been applied to obtain multiple local components in a dataset having healthy controls (Ct) and Sz, leading to the speculation that Sz may represent a set of eight distinct clinical disorders. The same methods were applied for the first time to imaging FA data to decipher subtypes in Sz (30).

**Figure 1:**
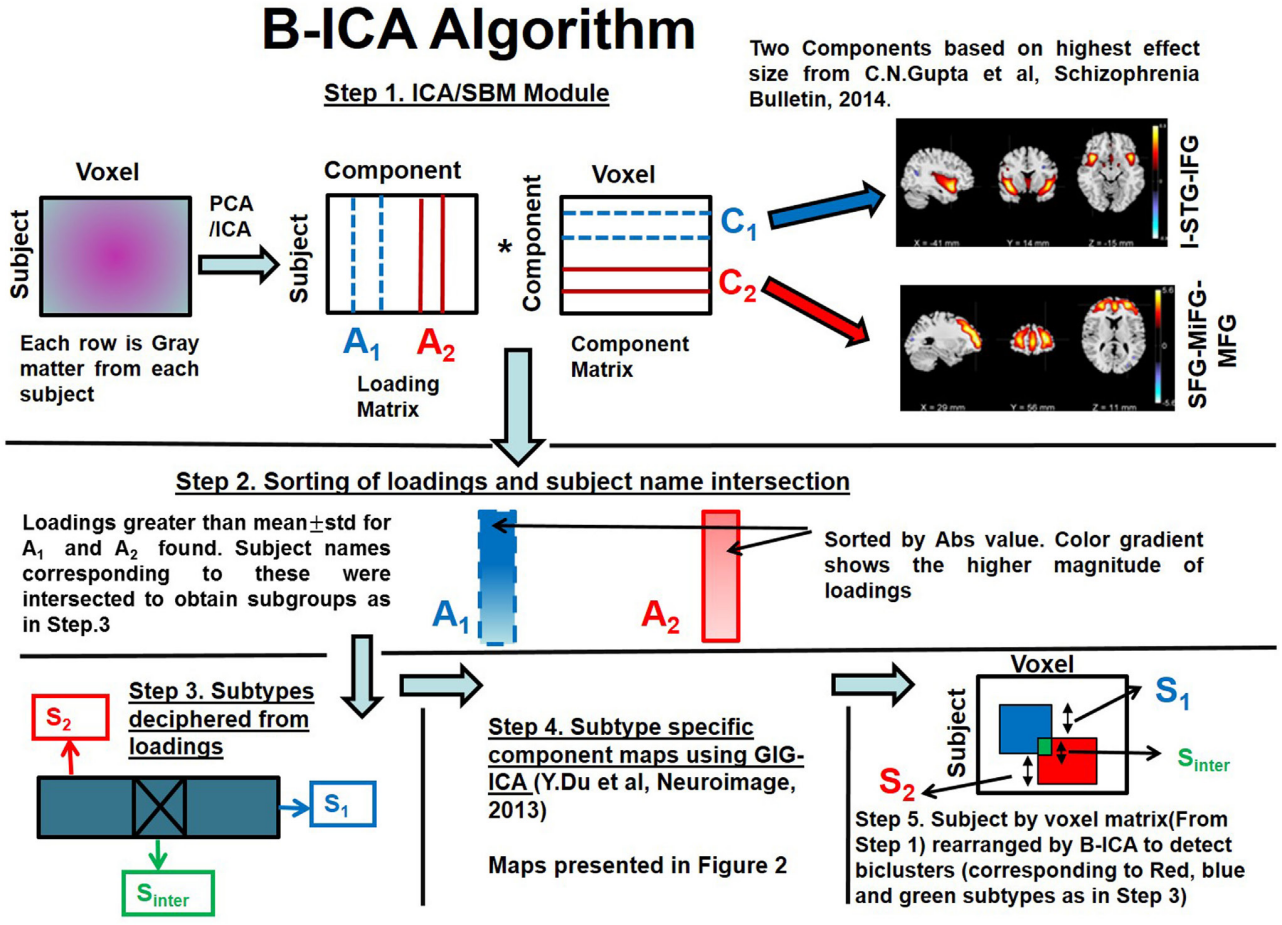
Biclustered Independent Component Analysis (B-ICA) framework illustrating the various steps to decipher subtypes.

Source-based morphometry is now an established multivariate technique which combines information across different voxels for imaging modalities to give spatial components (i.e., spatially connected regions) that differ between two groups rather than region of voxels (13). It is known sMRI varies to a lesser degree over time than clinical/cognitive symptoms. Hence, through this work, we propose a new methodology for subtyping patients with Sz from the reliable/stable GMC rather than doing a factor analysis on symptom scores as in most previous studies. To the best of our knowledge, this is the very first work which does not use healthy controls sMRI data during clinical subtyping. Recently, reliable replication of GMC components showing (Ct/Sz) diagnostic differences were assessed in the largest aggregated structural imaging dataset to date for Sz (11). We reported nine reliable components that showed diagnostic differences; seven had greater GMC and two had lower GMC in Sz than Ct (11). These components did not show relationship with clinical symptoms, when considered individually. We, therefore, decided to evaluate the relationship between symptoms and SBM loadings in subsets of subjects. These subsets were obtained by considering loadings from two components comprising regions of insula/superior temporal gyrus/inferior frontal gyrus (I-STG-IFG component) and superior/medial/middle frontal gyrus (SFG-MiFG-MFG component) which had high effect size and showed diagnostic differences from our previous work (11). Our method is outlined in Figure 1: following ICA/SBM, we hypothesized there exist subsets of Sz participants linked to specific symptoms with different neuroanatomical alterations on these components. Joint distribution of loadings from two components was exploited to obtain subsets which were then tested for association with clinical symptoms (steps 2 and 3 of Figure 1). This method can also be applied to other neuroimaging modalities and this accurate subtyping could provide reliable endophenotype (34, 35) for personalized drug development in Sz (36).

## Methods

### Participant Demographics and Clinical Measures

This work involved aggregating multisite datasets. Each dataset including diagnosis, age at time of scan, gender, symptom scores, duration of illness, and chlorpromazine equivalents (Cpz eqvt) medications when available, were shared by each participating group according to the sites protocol. Study-wise demographic info is presented in Table 1 and clinical information in Table 2. The majority of Sz were on antipsychotic medications, either typical, atypical, or a combination. All Sz were clinically stable at the time of scanning. The positive and negative syndrome scale (PANSS) is a clinical symptom scale used for measuring symptom severity of patients with Sz (37). It provides balanced representation of positive and negative symptoms and gauges their relationship to one another and to global psychopathology (37). A total of 382 Sz (mean age = 36.4, SD = 11.65, range: 18–64, 274 males/108 females) having PANSS information from three independent studies (one being multisite) formed the aggregated dataset, which totaled to nine scanning sites. As these are legacy data, the studies were collected separately in space and time, therefore inter-rater reliability across studies is also not available. However, the inter-rater reliability within multisite study was maintained [i.e., for FBIRN3 data: collection, training, and annual certification of the raters on standard patient interviews was done (38)]. More details regarding the datasets and their publications are available in the supplemental material (appendix 1) of our previous publication (11).

**Table 1:** Demographic information by study.

Study name	Schizophrenia (Sz) sample size	Schizoaffective disorder	Male/female	Age (mean ± SD)	Age (min–max)	Sites
FBIRN 3	179	Not available	136/43	39.22 ± 11.60	18–62	7
TOP	128	18	76/52	31.80 ± 08.90	18–62	1
COBRE	75	7	62/13	37.56 ± 13.50	18–64	1

**Table 2:** Clinical information by study.

Study name	PANSS positive mean ± SD	PANSS negative mean ± SD	PANSS general mean ± SD	Duration of illness (DOI) mean ± SD	% Reporting (DOI)	Cpz eqvt mean ± SD	% Reporting (Cpz eqvt)
FBIRN 3	15.55 ± 5.11	14.44 ± 5.50	27.90 ± 7.26	17.77 ± 11.30	98.30	1,068.3 ± 6,266.2	84.36%
TOP	14.60 ± 5.23	15.0 ± 6.78	27.80 ± 8.15	6.58 ± 5.63	97.54	Not available	Not available
COBRE	15.42 ± 4.86	14.76 ± 4.94	27.90 ± 8.63	15.42 ± 11.72	98.70	1,023.7 ± 1,422.2	98.67%

*PANSS, positive and negative syndrome scale; Cpz eqvt, chlorpromazine equivalents*.

All studies were collected under local IRB oversight and participants provided informed consent. The structured clinical interview for diagnosis for DSM-IV or DSM-IV-TR was used to confirm a diagnosis of Sz or schizoaffective disorder (SzAff) in few datasets. We do not consider inclusion of SzAff to be a significant source of variation since recent work has identified that structural differences between Sz and Szaff are similar (39). We regressed out site on PANSS general scores as it showed an effect, with other scores not exhibiting a site effect.

### Image Preprocessing

The scanning sites included 1.5 and 3 T scanners from various makes/models, collecting T_1_-weighted images using sagittal orientation and MPRAGE sequences as in Table 3. Using the methods presented in Ref. (11–13) images were normalized using a 12-parameter affine model to the 152 average T_1_ Montreal Neurological Institute template, resliced to 2 mm × 2 mm × 2 mm, and segmented into gray, white, and CSF images using the unified segmentation algorithm (7) of SPM5 (http://www.fil.ion.ucl.ac.uk/spm/software/spm5/). We used the same standard preprocessing pipeline from our previous studies (11–13). Outlier GMC images were identified based on correlations to both a study-specific template and an averaged GMC map. They were then visually checked, corrected, and re-segmented where possible, and removed in cases where correction was not possible. The sample sizes presented in Table 1 are those images which passed the quality assurance methods. Age, gender, and site were regressed out on the images voxelwise as these variables were not of interest (11). A full width half maximum Gaussian kernel of 10 mm was used to smooth the images prior to the VBM and SBM analyses as suggested in Ref. (10, 40).

**Table 3:** Scanner information by study.

Study name	Manufacturer, model, and field strength (*T*)	Sequence	Voxel size (mm)	Scanning orientation
FBIRN 3	Siemens Tim Trio (3)	MPRAGE	1.1 × 0.9 × 1.2	Sagittal
TOP	Siemens (1.5)	MPRAGE	1.33 × 0.94 × 1	Sagittal
COBRE	Siemens Tim Trio (3)	MPRAGE	1 × 1 × 1	Sagittal

### Biclustered Independent Component Analysis (B-ICA) Framework for Subtype Detection

We present the B-ICA framework pictorially and explain the method with reference to Figure 1. This framework is tuned for sMRI, but it can be applied to other neuroimaging modalities as well. It consists of:

(1) SBM decomposition on GMC matrix from patients with Sz only as in Eq. 1 (13)
  (1) $$X=A_{1}C_{1}+A_{2}C_{2}.....+A_{N}C_{N}$$
  where X stands for observed source matrix. *C*_1_, *C*_2_, …, *C_N_* are the underlying original sources or natural groupings and *A*_1_, *A*_2_, … *A_N_* are the corresponding loadings. We selected two components (*C*_1_ and *C*_2_) corresponding to I-STG-IFG and SFG-MiFG-MFG components (step 1 of Figure 1) from our previous work (11), which had the highest effect sizes.
(2) Loadings for the two selected components (*A*_1_ and *A*_2_) were sorted by absolute value as indicated by the gradient color change in (step 2 of Figure 1). Loadings greater than a statistical threshold (mean ± SD) for both components were found. Subject names passing this threshold from both components were then intersected to obtain subtype *S*_inter_.
(3) Subtypes were found as below (step 3 of Figure 1).
  *S*_inter_—subjects who are highly weighted on both *C*_1_ and *C*_2_;
  *S*_1_—subjects who are exclusively highly weighted on *C*_1_;
  *S*_2_—subjects who are exclusively highly weighted on *C*_2_;
(4) Subtype-specific components (step 4 of Figure 1) were then reconstructed with the subtype loadings using group information-guided ICA (GIG-ICA) algorithm, as it preserves independence of the components at subtype level (41).
(5) Subtypes along the subject dimension in the subject by voxel sMRI matrix after application of B-ICA is illustrated (step 5 of Figure 1).

This algorithm first finds subtypes using two component loadings (along the subject dimension). Then using the deciphered subtype loadings, a GIG-ICA step is performed to find subtype-specific components (along the voxel dimension). Since we achieve clustering in both subject and voxel dimensions, this is considered as biclustering. The algorithm effectively rearranges voxels inside a huge subject by voxel matrix (step 1 of Figure 1) to decipher overlapping biclusters (illustrated as red and blue squares in step 5 of Figure 1).

### Non-Parametric Testing of Clinical Symptoms between Deciphered Subtypes

The PANSS was considered and the positive (PP), negative (PN), and general (PG) clinical scores were summed. Being multisite data, we regressed out site effects on the summed scores, where present. For the identified subtypes *S*_1_. *S*_2_, we performed a Mann–Whitney test (*U*) (42) between their corresponding PP, PN, and PG scores as the distributions were not normal and due to their small sample sizes. Correlations between the ICA loadings of each subtype and the PP, PN, and PG scores were also calculated.

### Structural Network Connectivity (SNC)

To further elucidate the subtyping we also performed SNC analysis (43) for the identified groups. SNC is measured *via* the correlations obtained between the loadings of the two components in the inferred subtypes.

## Results

Independent of clinical subtyping, we first tested the association of loadings from both components with PANSS positive, negative, and general scores observing no significant association as presented in Table 4.

**Table 4:** Correlations between component loadings across all participants.

Component/PANSS	Positive (*R*, *p*)	Negative (*R*, *p*)	General (*R*, *p*)
I-STG-IFG	*R*(380) = 0.02, *p* = 0.59	*R*(380) = −0.07, *p* = 0.13	*R*(380) = 0.02, *p* = 0.58
SFG-MiFG-MFG	*R*(380) = 0.06, *p* = 0.23	*R*(380) = −0.08, *p* = 0.09	*R*(380) = 0.05, *p* = 0.29

*PANSS, positive and negative syndrome scale*.

**Figure 2:**
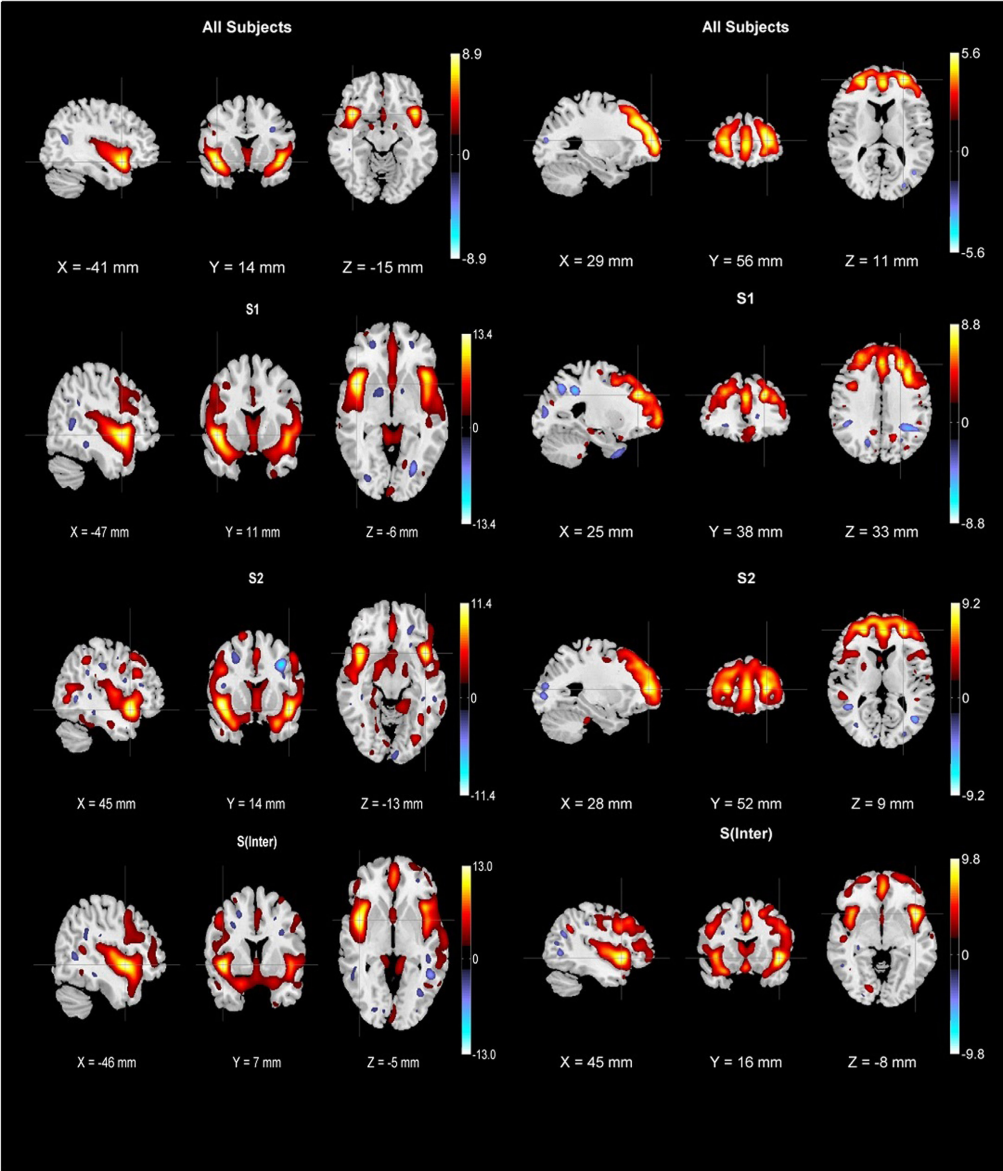
First row: group components for 382 schizophrenia subjects (column one is insula/superior temporal gyrus/inferior frontal gyrus component while column two is superior frontal gyrus/middle frontal gyrus/medial frontal gyrus component). Subtype-specific components were reconstructed using biclustered independent component analysis and group information-guided ICA. Second row: *S*_1_ subtype components (65 subjects), third row: *S*_2_ subtype components (62 subjects), fourth row: *S*_inter_ subtype components (53 subjects). All components were thresholded at |*z*| > 2.5 and cross hairs indicate the maximum voxel.

**Figure 3:**
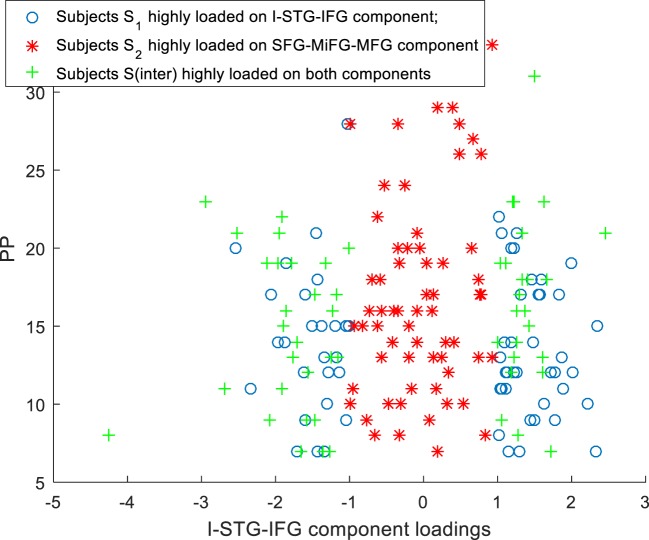
Scatter plots for subtypes *S*_1_, *S*_2_, *S*_inter_ I-STG-IFG component loadings Vs PP.

After applying the B-ICA algorithm as in Figure 1 on the GMC matrix of 382 Sz, we obtained two exclusive subtypes *S*_1_ (65 subjects highly weighted on only I-STG-IFG component), *S*_2_ (62 subjects highly weighted on only the SFG-MiFG-MFG component), and one intersecting group *S*_inter_ (53 subjects highly weighted on both components). The group and subtype-specific reconstructed components obtained using GIG-ICA are shown in Figure 2. We observed variations in reconstructed components for different subtypes, when compared to the group component (considering all 382 Sz subjects). The reconstructed subtype-specific components showed subtle variations in several regions, when compared with the group components as in Figure 3. For the I-STG-IFG component (column 1 of Figure 2) the subtypes (*S*_2_ and *S*_inter_) components showed additional regions of precentral gyrus, anterior cingulate and medial frontal gyrus, while for the SFG-MiFG-MFG component (column 2 of Figure 2), s(inter) showed regions of cingulate gyrus, middle temporal gyrus and inferior frontal gyrus.

### Association of Subtypes *S*_1_, *S*_2_, and *S*_inter_ with PP, PN, and PG

**Figure 4:**
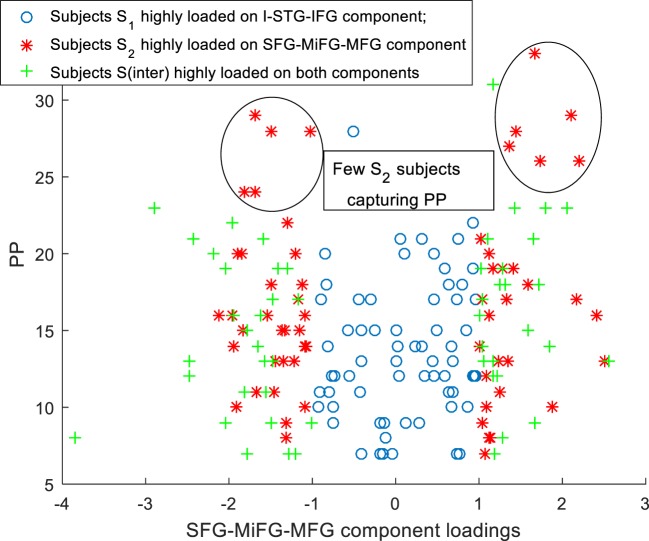
Scatter plot for subtypes *S*_1_, *S*_2_, *S*_inter_ SFG-MiFG-MFG component loadings Vs PP.

Scatter plots of subtype loadings *S*_1_, *S*_2_, *S*_inter_ in components *A*_1_, *A*_2_ Vs PP is depicted in Figures 3 and 4, respectively. PP scores in subtypes *S*_1_ (mean = 13.68) and *S*_2_ (mean = 16.74) showed a significant difference with a Wilcoxon rank sum test = 3,954 (*n*_1_ = 65, *n*_2_ = 62, *p* = 0.006). We observed few subjects in subtype *S*_2_ capturing the high PP (circled in Figure 4). No significant differences in PN and PG scores were observed between these subtypes *S*_1_ and *S*_2_. Subtype demography/clinical information is included in Table 5.

**Table 5:** Demography/clinical information across all subjects and subtypes.

	All 382 schizophrenia (Sz)	*S*_1_ (65 Sz)	*S*_2_ (62 Sz)	*S*_inter_ (53 Sz)
PP	15.21 ± 5.11	13.68 ± 4.46	16.74 ± 6.21	15.47 ± 5.26
PN	14.69 ± 5.86	13.86 ± 5.67	14.74 ± 5.39	14.64 ± 5.43
PG	27.91 ± 7.83	27.64 ± 7.44	28.24 ± 7.25	27.79 ± 9.25
Age	36.4 ± 11.65	36.09 ± 12.24	35.25 ± 10.81	35.64 ± 12.15
Gender	274 Males/108 females	49 Males/16 females	38 Male/24 females	40 Males/13 females

We also examined the associations of *S*_inter_ loadings in both *A*_1_, *A*_2_ with PP, PN, and PG. We observed a trend level correlation of *R*(51) = 0.25, *p* = 0.07 for the loadings of *S*_inter_ in *A*_2_ with PG symptoms.

### Structural Network Connectivity

The SNC between I-STG-IFG and SFG-MiFG-MFG component loadings for the three subtypes showed varying strengths of connectivity as follows: *S*_1_ subtype [*R*(63) = 0.51, *p* = 1.36e-5], *S*_2_ subtype [*R*(60) = 0.67, *p* = 1.67e-9], and *S*_inter_group [*R*(51) = 0.93, *p* < 0.00001].

## Discussion

This work presents a novel data-driven framework called B-ICA to unearth subtypes having complex biomarkers from neuroimaging data, by considering patients with Sz only. B-ICA applied here on a GMC helps to map the hidden latent relationship between the subset regions of GMC for a subset number of subjects and the clinical scores. This work tries to tackle the challenging task of subtyping patients with Sz without considering Ct. Our viewpoint is similar to the recent theory-driven systematic study, which identified two subtypes in patients with Sz using neuropsychological battery, assessment of clinical symptoms, neurological soft signs, morphogenetic anomalies, smell identification, and measurement of event-related potentials (44). The hypothesis that neuropsychiatric disorders are a result of combination of alterations with varying directionalities in different parts of the brain, is gaining acceptance (31). In support of this, numerous studies in Sz have reported GMC/GMV deficits throughout the brain with the areas of I-STG-IFG component (11, 35) being most severely and consistently affected in Sz. Larger basal ganglia volume (45), striatal gray matter density (35), GMC in cerebellum/brainstem and putamen (46) have also been reported in Sz, compared to Ct group. Most of these studies look at global differences between Ct and Sz, without considering the differences in clinical symptoms within the Sz cohort. With such an analytic viewpoint, low discriminative components between patients with Sz are often missed in a high dimensional neuroimaging dataset, which we managed to decipher in this work. The ICA components analyzed in this work also showed maximum group difference between Ct and Sz in our previous work (11).

**Figure 5:**
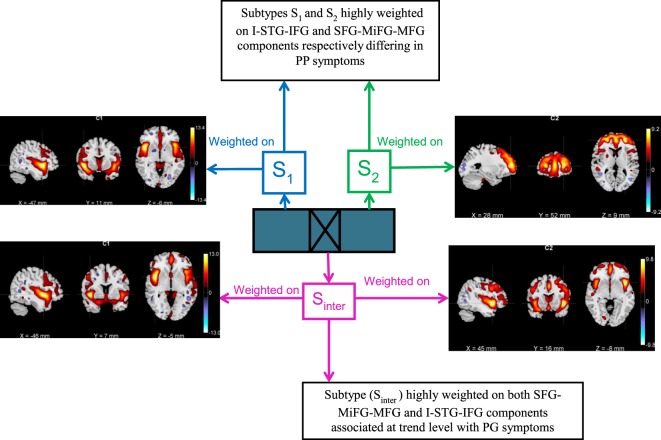
Bird’s eye view of the subtype associations with positive and negative syndrome scale clinical symptoms obtained using biclustered independent component analysis.

A bird’s eye view of the associations between various subtypes and the clinical symptoms obtained using B-ICA is shown in Figure 5. We observed a complex biomarker (i.e., in terms of deviations from mean GMC on two components) for subtypes *S*_1_ and *S*_2_. The *S*_inter_ group had higher GMC on both the components. *S*_2_ included few Sz subjects with high positive symptom severity (Figure 4), driving the difference in PP between *S*_1_ and *S*_2_; this difference include both positive and negative loadings on the SFG-MiFG-MFG component. It is not simply “more” or “less” of that GMC component that predicts the increased positive symptoms; but it is the deviation from the mean values, as shown in Figure 4. Taken together, this could effectively mean that distinct subtypes of Sz are characterized by varying trends of GMC abnormalities in different regions of the brain. Recently subgroups of Sz differing in PANSS symptoms was also reported in a resting state cerebral blood flow work (47).

These results also suggest that the group of subjects with more extreme weightings on SFG-MiFG-MFG component who also show a weaker weighting in the I-STG-IFG component, is likely to include subjects with greater positive symptoms. While structural interactions between these two GMC components are highly speculative, these are also areas that have been related volumetrically to psychotic symptoms in the prior literature, particularly reduced superior frontal volume with positive symptoms across the psychosis spectrum (48) and in non-clinical samples (49). The insular cortex is both functionally and structurally affected in Sz, and as part of the salience network may be playing a fundamental role in the development of psychosis (50, 51). The particular component we find of not just decreased GMC in one area, but a deviation from the norm in the SFG-MiFG-MFG component, while having an average GMC expression in the I-STG-IFG component, makes these participants a promising group for future more clinically oriented study.

Our approach does not require any *a priori* knowledge or assumption on the number of biclusters in the subject by voxel matrix, except for the simple statistical threshold to search for subtypes. In contrast to clustering techniques like k-means (52) and hierarchical clustering (53) that find global components based on clinical or cognitive symptoms which are characterized by heterogeneity, B-ICA’s unique data-driven approach enables detection of reliable hidden subtyping from the reliable neuroimaging data. It untangles both overlapping and non-overlapping biclusters based on the inherent properties of GMC data, which in turn is dependent on the spectrum of Sz recruited. These biclusters (i.e., subset of subjects and voxels) linked to specific clinical symptoms could serve as a possible endophenotype for Sz (on replication in future studies) to indicate regions which are affected by common factors like genetics or disease progression (34). We agree, that these sMRI components are not marked endphenotypes in themselves at this point. A much more detailed study of their relationships across diagnostic boundaries, within families, and changes over the development of the illness would be required before their potential as an endophenotype could be determined. The point of this analysis is to introduce the idea of complex combinations of these measures being related to symptoms and patient subgrouping, that would need to be examined with regards to prognosis.

There are a few limitations in this work. First, all the Sz were on antipsychotic medications, and in many datasets, the amounts/history of dosage are not recorded. The availability of standardized Cpz eqvt on all Sz would have made the analysis stronger. Cognitive symptom tests were not considered due to its non-availability across datasets; however, conceptual opportunities in future do exist for the use of RDoc (Research Domain criteria). We also did not have access to electrophysiological data, though that would have been a strength. It is also true that other components (i.e., brain regions) might be relevant to psychotic symptoms and this would require clustering of multiple components, which we plan to investigate in future. We did control for site effects on PANSS scores; however, there could exist heterogeneity due to differences in clinicians rating. The use of biclustering for neuroimaging analyses is relatively new, so it is envisaged this work will stimulate further research. Additionally, future work should be done to replicate these results.

## Ethics Statement

This study was performed on legacy datasets that were shared by and with the coauthors for the purpose of this secondary analysis. All data were de-identified and no subjects were recruited for the purpose of this paper. Thus this secondary analysis does not constitute a study which requires IRB oversight.

## Author Contributions

CG, EC, SR, VC, and JT developed the B-ICA algorithmic framework and contributed to numerous discussions. CG performed the data analysis. CG, VC, and JT interpreted the results and wrote the paper. All other authors contributed datasets to this work.

## Conflict of Interest Statement

The authors declare that the research was conducted in the absence of any commercial or financial relationships that could be construed as a potential conflict of interest.
